# Clustering matrix-object data by correlational structure as proxy causal signals

**DOI:** 10.1038/s41598-026-61243-w

**Published:** 2026-07-10

**Authors:** Ziheng Qi, Liqin Yu, Junfei Li

**Affiliations:** School of Information Engineering, Beijing Institute of Graphic Communication, No. 1 Xinghua Street (Section 2), Beijing, 102600 China

**Keywords:** Matrix-object data, Clustering, Correlational structure, Proxy causal signals, Computational biology and bioinformatics, Mathematics and computing, Physics

## Abstract

Matrix-object clustering addresses samples with multiple records per object. Many existing methods overlook within-object dependencies, which reduces interpretability and mixes heterogeneous regimes. We propose a clustering approach that uses intra-object correlational structure as a proxy for causal signals to separate regimes prior to any formal causal discovery. Each object is transformed into a rank-based correlation representation, enabling standard distance-based clustering while preserving interpretability. On synthetic and real-world datasets, the method yields stable, interpretable clusters that reduce regime mixing. We emphasize the boundary that correlation does not imply causation; correlational patterns are used only as proxy signals under the stated assumptions.

## Introduction

Clustering is an unsupervised learning technique used to divide objects into several groups such that objects within the same group are highly similar to each other, and objects in different groups are less similar^1–7^. Interpretable clustering emphasizes that algorithms should not only group data into clusters but also provide reasons for the grouping^8–12^. In the medical field, researchers may need to understand why certain patients are grouped in the same cluster to better understand disease types or treatment responses^13–15^.

Matrix-object clustering refers to methods applied to data containing multiple matrix-objects, each with more than one record. The number of records may vary across matrix-objects^16^. Such data are common across many real-world domains. In text clustering, each document can be viewed as a matrix-object; in image processing, a local region of an image can be treated as a matrix-object for analysis. However, matrix-object data may need to be processed to satisfy the input requirements of traditional algorithms.

In the realm of matrix-object data clustering, Cao and Yu have, in recent years, successively introduced partitioning clustering algorithms tailored for categorical and numeric matrix-object data, respectively, which filled the gap in clustering algorithms designed for matrix-object data^17,18^. Targeting the one-to-many and evolutionary characteristics of matrix-object data, Yu proposed a weighted partitioning clustering algorithm and a hierarchical multi-granularity evolving clustering algorithm specifically for matrix-object data^16^.

**Fig. 1:**
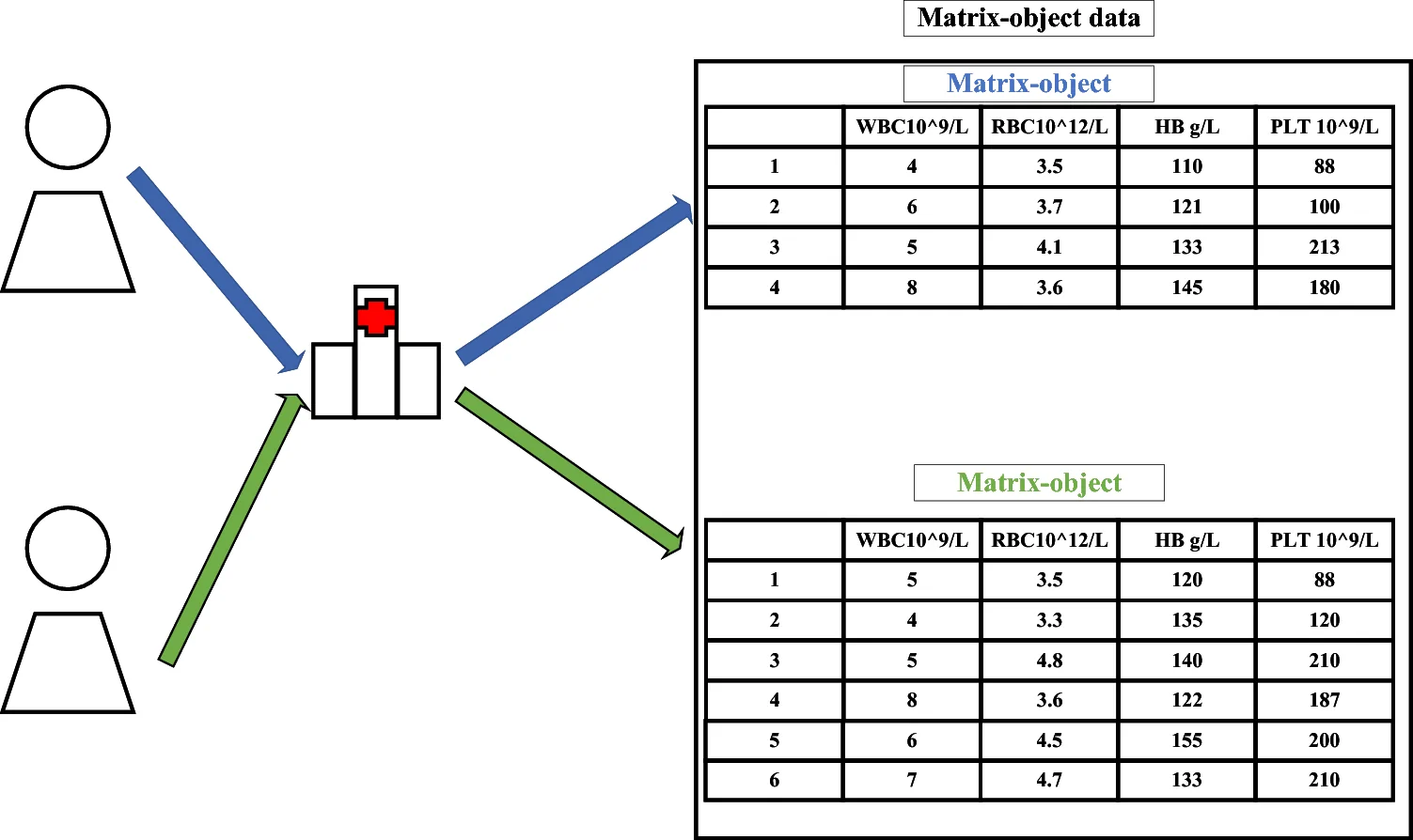
Example of matrix-object data in medical research.

In medical research, matrix-object data are ubiquitous. In Fig. 1, two patients visit a hospital four times and six times over a period of time, respectively, for routine blood tests that measure White Blood Cell Count (WBC), Red Blood Cell Count (RBC), Hemoglobin (Hb), and Platelet Count (PLT). The examination results of each patient form a matrix-object, and multiple matrix-objects are combined into a matrix-object dataset. Such data are widely observed in medical research.

Recent work combining causality with clustering includes the following. Alex Markham (2020)^19^ proposed a kernel clustering method that maps low-dimensional single-instance data to a higher-dimensional space via kernel functions before clustering. Huang and Zhang (2019)^20^ defined a class of shared causal models that facilitate mechanism-based clustering from a causal perspective. Although these methods consider causality in the clustering process, the data types targeted by the algorithms are not matrix-object data.

In this paper, we propose a clustering method for matrix-object data from a correlational perspective under stated assumptions (proxy signals). The contributions are summarized below:

1. We overcome limitations in matrix-object clustering by proposing a method that finds groups with different correlational structures between features; under the stated assumptions, these structures serve as proxy signals for differences in causal skeletons at the clustering stage.

2. We propose a new representation of matrix-objects and conceptualize a framework for matrix-object clustering that captures intra-object correlational structure and yields interpretable results.

3. Our proposed algorithm models different motion states with distinct regimes between features in real-world datasets to achieve accurate identification.

In Section “Preliminaries”, we introduce the relevant knowledge needed to understand our work. In Section "The proposed algorithm", we propose the correlational-structure clustering method to cluster the matrix-object data according to the correlational structure between the features of matrix-objects. Section “Experiments” presents the experimental results of the proposed algorithm, along with comparisons with other clustering algorithms. Section "Conclusions, discussions, and future work" summarizes the contributions and provides directions for future research.

## Preliminaries

In this section, we introduce the related literature needed to understand our work including the Structural Causal Model, Time Series Model, and the distinction between correlation and causation (correlation does not imply causation).

### Structural Causal Model (SCM)

#### Definition 1

The modern definition of causality is that if changing *X* leads to a change in *Y* while keeping all other factors constant, then *X* is considered a cause of *Y*, written as *X → Y*. The causal structure between features is commonly represented by a Structural Causal Model (SCM). The SCM is a model defined on a set of variables that represents causal relationships through a Directed Acyclic Graph (DAG).

**Fig. 2:**
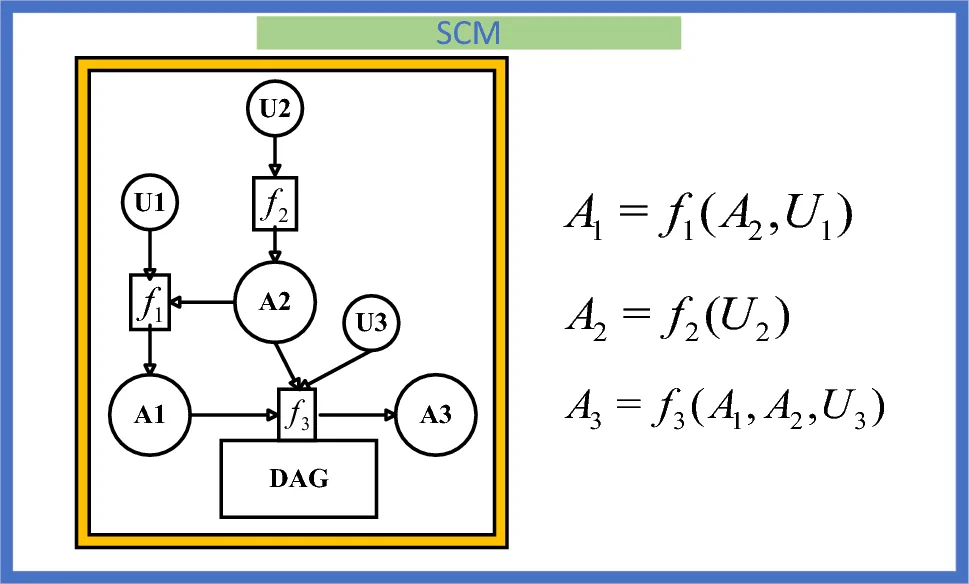
Structural causal model.

The SCM consists of a series of assignment rules that describe how to determine the value of each variable through other variables. For variables $A_1,\ldots ,A_m$, the SCM can be represented as a set of equations, where each equation defines how a variable is determined based on its parent set (i.e., the set of variables that have a direct impact on it) and each node $A_j$ corresponds to an exogenous variable $U_j$ that is not observed^21–23^, as shown in Fig. 2.

### Time Series Model (TSM)

#### Definition 2

A time series model is a mathematical model used to describe patterns of change in multiple variables over time. In time series causal research, a common assumption is that the data is generated by a Structural Vector Autoregression (SVAR) process. For a multivariate time series $A(t) = [A_1(t), \ldots , A_m(t)]$ defined at discrete time steps, where *m* is the number of variables and *t = 1, … , T* represents the time steps, each variable $A_j(t)$ can be expressed as:

1 $$\begin{aligned} \ A_j(t) = f_j(\text {PA}_j(t - 1), N_j) \end{aligned}$$

We use these SCMs as reference regimes to assess whether the proposed method correctly separates matrix-objects into homogeneous groups prior to any causal discovery; we stress that correlational clustering is not, by itself, causal identification. $f_j$ is the structural function, $\text {PA}_j$ represents the parent nodes of $A_j$ (i.e., the variables that causally influence it), and $N_j$ is the exogenous noise term.

**Fig. 3:**
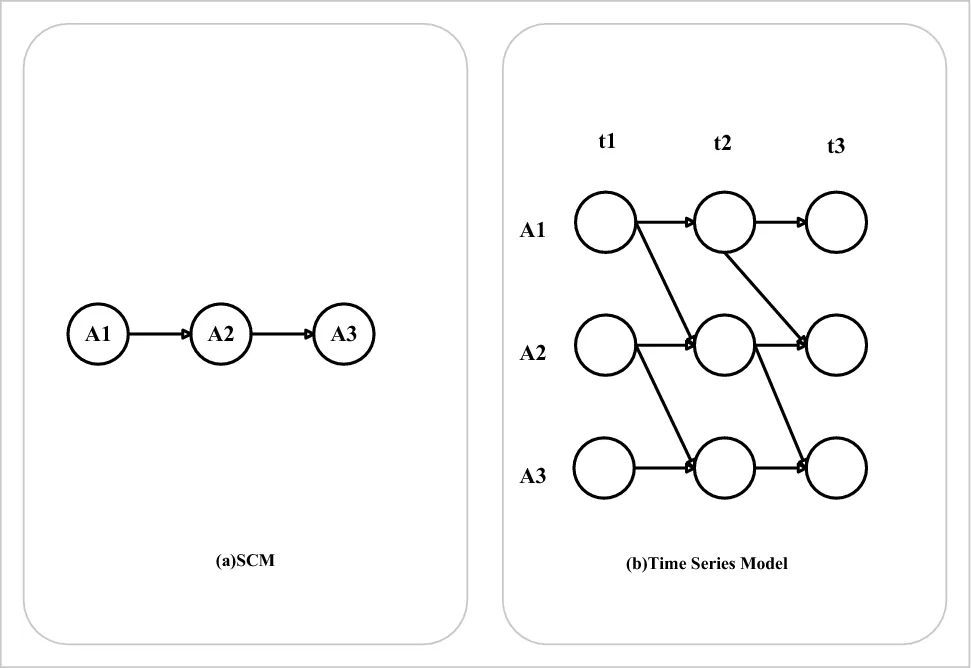
Time series model and SCM.

The difference between SCM and the Time Series Model^24^ is shown in Fig. 3. In Fig. 3 (b) the three values in each row represent the values of each attribute ($A_1$, $A_2$, $A_3$) at the three time points $t_1$, $t_2$, and $t_3$. As can be seen in the figure, the Time Series Model (TSM) is a special case of SCM under time series data, such as the value of $A_2$ at $t_2$ depends not only on the value of $A_2$ at $t_1$, but also on the value of $A_1$ at $t_1$.

### Correlation vs. causation: correlational patterns as proxy signals

Correlation and causality are two fundamental concepts in statistics and epidemiology that describe the relationship between variables. Correlation refers to the statistical association between two variables, manifesting as a quantifiable linear or nonlinear relationship, the strength which can be measured by a correlation coefficient. The presence of correlation does not imply causality, as it may be influenced by confounding variables or mere coincidence. In contrast, causality involves a more rigorous logical relationship, where a change in one variable (the cause) directly leads to a change in another variable (the effect).

**Scope and assumptions.** To use correlational structures as proxy signals at the clustering stage in a logically rigorous manner, we rely on monotone mechanisms, independent noise, the absence of hidden confounding for compared pairs, and stationarity with sufficient samples. These conditions are enumerated as Assumptions 1–3 and formalized in Proposition 1 in Section "A new representation of matrix-object". Under these assumptions, correlational patterns help distinguish heterogeneous regimes but do not, by themselves, establish causality; formal causal discovery must be conducted after clustering.

## The proposed algorithm

In this section, we first introduce the definition of matrix-object data, then give the motivation for developing the new algorithm, next propose a new matrix-object representation that captures the correlational structure between attributes of matrix-objects, and finally present the correlational-structure clustering algorithm.

### Matrix-object data

#### Definition 3

Let $\textbf{X}=\{X_1,X_2,\ldots ,X_n\}$ denote a matrix-object dataset that contains *n* matrix-objects and is described by *m* features $\{A_1,A_2,$ $\ldots ,A_m\}$. The *i*th matrix-object $X_i$ $(1\le i\le n)$ contains $r_i$ *m*-dimensional feature vectors. $r_i$ may be different for different matrix-objects. The matrix-object $X_i$ is written as

$$\begin{aligned} X_i= \left( \begin{array}{llcl} v_{i11} & v_{i12} & \cdots & v_{i1m}\\ v_{i21} & v_{i22} & \cdots & v_{i2m}\\ \vdots & \vdots & \ddots & \vdots \\ v_{ir_{i}1} & v_{ir_{i}2} & \cdots & v_{ir_im} \end{array} \right) . \end{aligned}$$

### Motivation

**Fig. 4:**
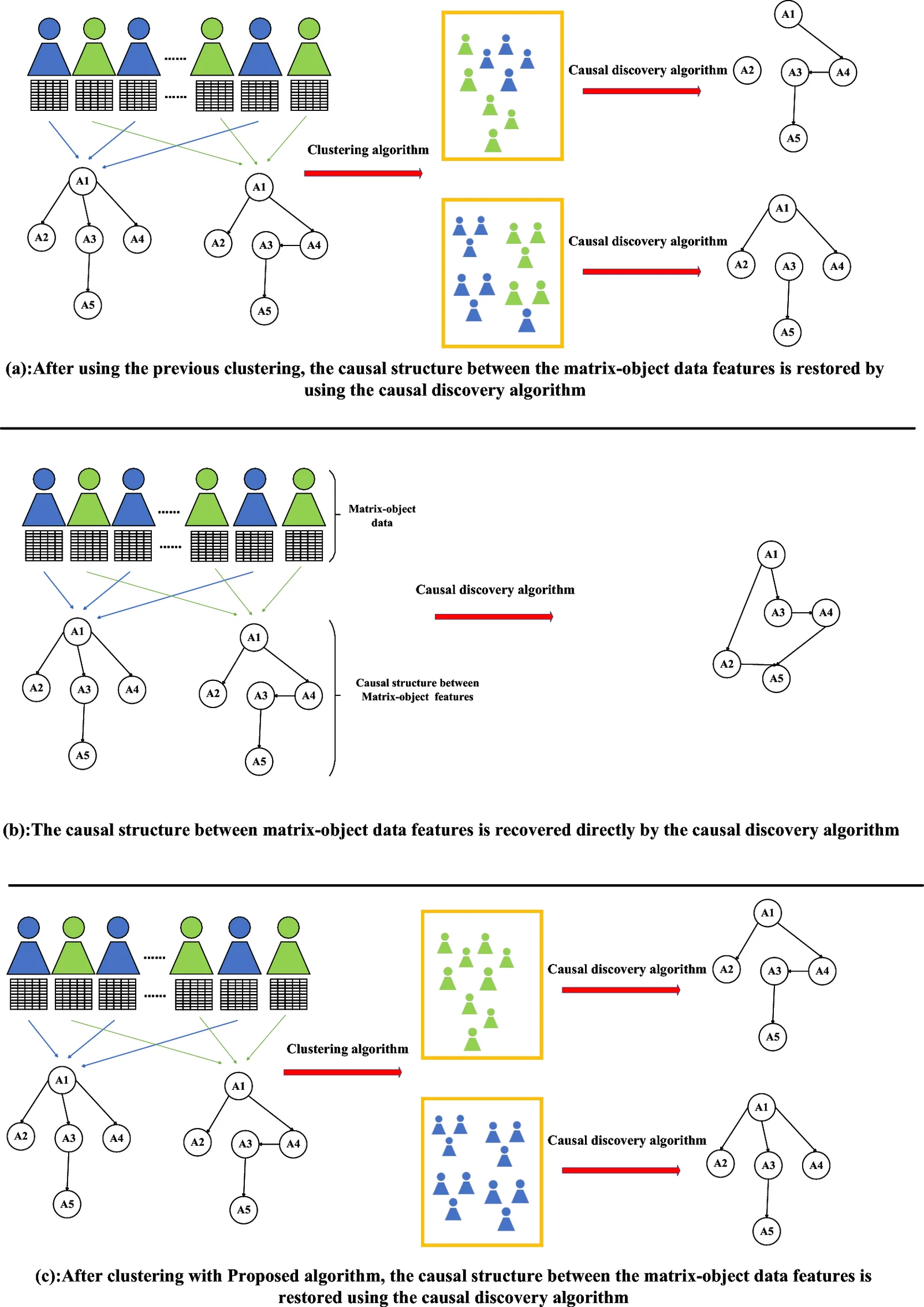
Motivation: cluster matrix-objects by intra-object causal structure to avoid mixing heterogeneous regimes.

Existing matrix-object data clustering methods typically ignore the causal structure within each object, which leads to block partitioning that mixes heterogeneous dependency patterns. Discovering causal graphs directly from unpartitioned or incorrectly partitioned data can yield inaccurate structures. Fig. 4 illustrates this issue: matrix-objects sharing similar causal patterns are marked with identical colors, where blue and green represent distinct causal structures. Conventional clustering methods often group samples with different causal structures into the same cluster. In Fig. 4 (a), each cluster contains both green and blue samples. Directly applying causal discovery algorithms to unclustered matrix-object data would yield a single structure Fig. 4 (b), whereas the matrix-object data may inherently contain multiple regimes. Moreover, the derived causal graphs may deviate from the true causal skeletons if heterogeneous regimes are mixed. To address these limitations, we propose a clustering algorithm that groups matrix-objects by their intra-object causal structure. As demonstrated in Fig. 4 (c), this method successfully clusters green and blue samples into distinct groups with their respective causal patterns preserved. Consequently, objects sharing similar causal structures are grouped together while those with differing structures are separated. This approach enhances downstream causal discovery by first isolating homogeneous regimes. We emphasize that correlational structure is used as a proxy signal under stated assumptions (Section "Correlation vs. causation: correlational patterns as proxy signals"), not as a proof of causality.

### A new representation of matrix-object

To cluster matrix-object data from a causal perspective, we propose a new representation of matrix-objects, then cluster matrix-object data to find groups. The new way of representing matrix-objects leverages intra-object monotone dependency captured by rank-based correlation. Spearman’s rank correlation coefficient is a nonparametric statistical measure designed to assess the strength and direction of the monotonic relationship between two variables^25–30^. As a matrix-object has multiple records, inspired by Spearman’s rank correlation coefficient, we can take each matrix-object as a dataset, and define the correlation between attributes for each matrix-object as follows.

#### Definition 4

Given a matrix-object $X_i$ described by *m* attributes $\{A_1, A_2, \ldots , A_m\}$, the correlation between attributes $A_s$ and $A_t$ for the matrix-object $X_i$ is defined as follows.

2 $$\begin{aligned} CR_{X_i}(A_s,A_t) = 1 - \frac{6 \sum _{p=1}^{r_i} (R(v_{ips})-R(v_{ipt}))^2}{r_i((r_i)^2 - 1)}, \end{aligned}$$

where $r_i$ is the number of records in the matrix-object $X_i$. $v_{ips}$ and $v_{ipt}$ denote the *p*-th record’s values of $X_i$ on attributes $A_s$ and $A_t$, respectively. $R(v_{ips})$ denotes the rank of the value $v_{ips}$ in the matrix-object $X_i$ on attribute $A_s$. $R(v_{ips})$ is defined as follows.

3 $$\begin{aligned} R(v_{ips}) = \sum _{q=1}^{r_i}g(v_{ips},v_{iqs}), \end{aligned}$$

where $g(v_{ips},v_{iqs})$ is an indicator function defined as follows.

4 $$\begin{aligned} g(v_{ips},v_{iqs}) = {\left\{ \begin{array}{ll} 1, & \text { if } v_{ips}\le v_{iqs} \\ 0, & \text { if } v_{ips}> v_{iqs}\end{array}\right. }. \end{aligned}$$

By definition, the rank of $v_{ips}$ equals the number of entries in $X_i$ on $A_s$ that are greater than or equal to $v_{ips}$. Thus, the maximum has rank $r_i$ and the minimum has rank 1.

In what follows, we specify assumptions under which correlational patterns can serve as proxy signals for differences in causal skeletons, and highlight failure modes.

#### Assumption 1

(*Monotone mechanisms and independent noise*) Data within each matrix-object is generated by a structural causal model (SCM) of the form $A_j(t) = \sum _{r \in PA_j} f_{jr}(A_r(t-1)) + N_j$, where every $f_{jr}$ is monotone in its argument(s), and noise terms $N_j$ are mutually independent and independent of parents.

#### Assumption 2

(*No hidden confounding along compared pairs*) For any pair $(A_s, A_t)$ considered within an object, there are no unobserved common causes that induce spurious association beyond the modeled parent relations; equivalently, $(A_s, A_t)$ are d-separated conditional on parents in the true SCM when no direct or ancestral link exists.

#### Assumption 3

(*Stationarity and sufficient samples*) The intra-object records are identically distributed across time/instances, and $r_i$ is sufficient for rank-based correlation to reliably reflect monotone dependence.

#### Proposition 1

(Proxy signal under Assumptions 1–3) Under Assumptions 1–3, if $A_s$ is an ancestor of $A_t$ via a path composed of monotone mechanisms, then $\textrm{sign}(\,CR_{X_i}(A_s, A_t)\,)$ equals the product of signs along that path and $|CR_{X_i}(A_s, A_t)|> 0$ with high probability as $r_i$ grows. If $A_s$ and $A_t$ are d-separated (no causal path and appropriate conditioning), then $CR_{X_i}(A_s, A_t) \rightarrow 0$ as $r_i \rightarrow \infty$. This justifies using correlational structure as a proxy to distinguish heterogeneous causal skeletons at the clustering stage, and does not prove causality.

According to Eq. (2), the correlation between any two attributes can be computed for each matrix-object. Because all matrix-objects share the same attribute space, each can be represented by an *m× m* correlation matrix, which facilitates distance computation even when record counts differ across objects. Each matrix-object provides multiple values per attribute, forming column vectors. Per Eq. (2), the closer two ranked column vectors align, the stronger the monotonic dependence between the corresponding attributes. This captures the intra-object correlational structure. Finally, we take the lower triangle of the *m× m* matrix to form a row vector and use it as the new representation of the matrix-object.

Based on the above assumptions and Proposition 1, we now formalize the representation construction in Algorithm 1.

**Algorithm 1:**
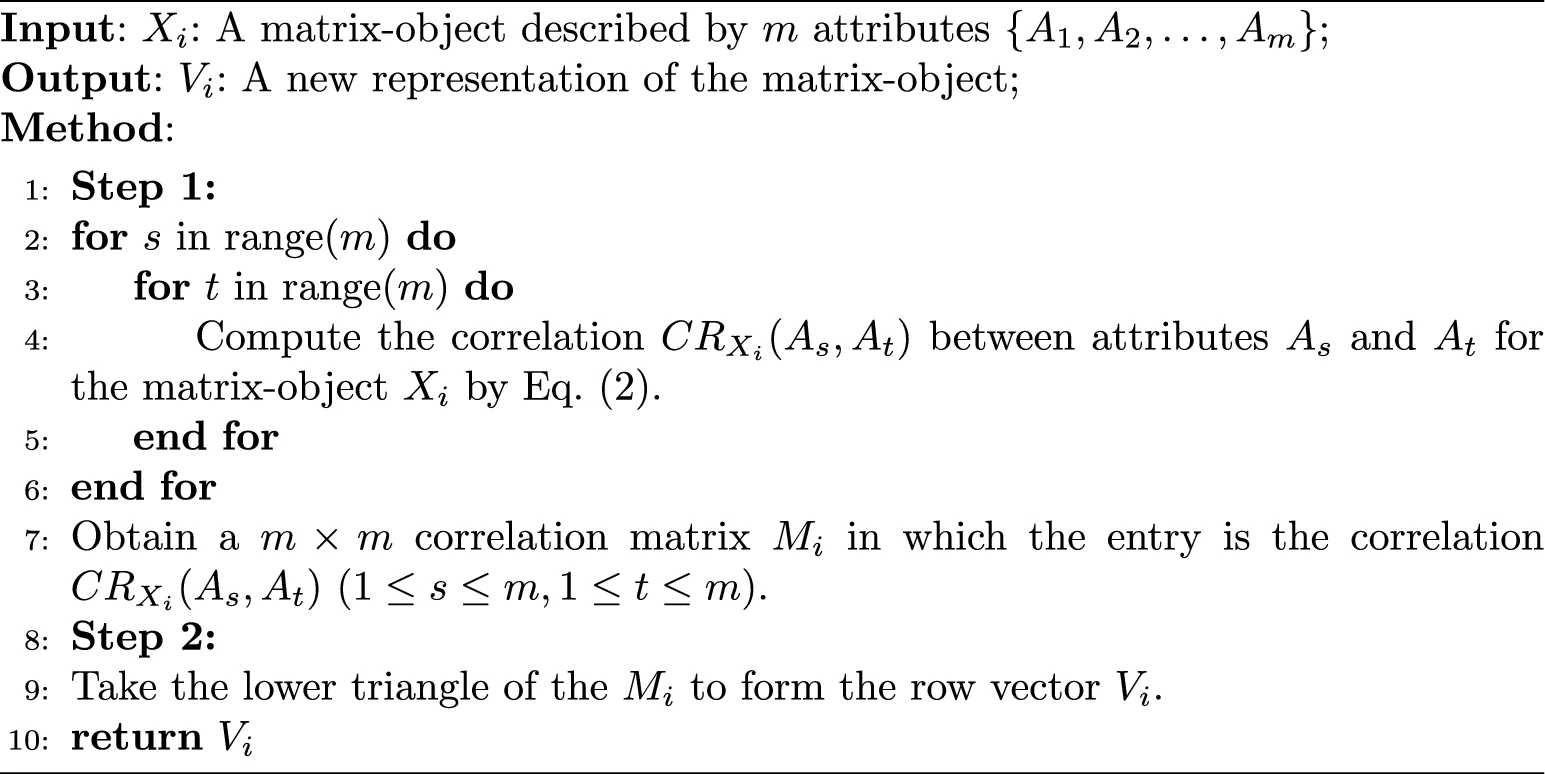
A new representation of matrix-objects based on intra-object rank correlations

An example of obtaining the representation of matrix-object using Algorithm 1 is as follows.

#### Example 1

Given two matrix-objects $X_1$ and $X_2$ described by five attributes $\{A_1, A_2, A_3, A_4, A_5\}$, they contain 10 and 6 records respectively. The matrix-objects are shown as follows. We can obtain the correlation among these attributes for $X_1$ and $X_2$ respectively. The algorithm systematically evaluates the pairwise correlations, starting with the correlation between $A_1$ and $A_2$, followed by the correlation between $A_1$ and $A_3$, and continuing in this manner until all pairwise combinations are assessed. This results in two symmetric *5 × 5* matrices $M_1$ and $M_2$ as follows.

$$\begin{aligned} X_1 = \begin{bmatrix} 0.37209543 & 0.22238038 & 0.89369444 & 0.53634979 & 0.52641806 \\ 0.53411713 & 0.53987284 & 0.71887145 & 0.82358972 & 0.24061252 \\ 0.43536933 & 0.77244662 & 0.22478771 & 0.04719425 & 0.770899 \\ 0.46507724 & 0.78508641 & 0.76094501 & 0.27642235 & 0.83665166 \\ 0.86615194 & 0.5538369 & 0.90243819 & 0.66119711 & 0.87835641 \\ 0.73674086 & 0.79798201 & 0.26071158 & 0.3834469 & 0.01120409 \\ 0.28330186 & 0.06342321 & 0.79568949 & 0.97057859 & 0.6511153 \\ 0.95583046 & 0.00422613 & 0.42787462 & 0.00650184 & 0.36311409 \\ 0.42169132 & 0.9946209 & 0.29395417 & 0.06664816 & 0.05111619 \\ 0.76180845 & 0.13809413 & 0.24704532 & 0.32868434 & 0.31695849 \end{bmatrix} \end{aligned}$$

$$\begin{aligned} X_2 = \begin{bmatrix} 0.01655179 & 0.01493201 & 0.43878085 & 0.75085699 & 0.80297675 \\ 0.53904316 & 0.27571161 & 0.50114875 & 0.11110489 & 0.62428887 \\ 0.25630469 & 0.30224269 & 0.45729795 & 0.62457314 & 0.56342334 \\ 0.08803228 & 0.428769 & 0.84124892 & 0.76233495 & 0.08566309 \\ 0.69294117 & 0.79054193 & 0.57156748 & 0.24812717 & 0.86160824 \\ 0.50024662 & 0.52477767 & 0.0787616 & 0.68167697 & 0.41391842 \end{bmatrix} \end{aligned}$$

$$\begin{aligned} M_1 = \begin{bmatrix} 1.0 & -0.16363636 & -0.15151515 & -0.28484848 & -0.04242424 \\ -0.16363636 & 1.0 & -0.23636364 & -0.18787879 & -0.18787879 \\ -0.15151515 & -0.23636364 & 1.0 & 0.6 & 0.49090909 \\ -0.28484848 & -0.18787879 & 0.6 & 1.0 & 0.06666667 \\ -0.04242424 & -0.18787879 & 0.49090909 & 0.06666667 & 1.0 \end{bmatrix} \end{aligned}$$

These matrices summarize monotonic associations between attributes. We take the lower-triangular entries of $M_1$ and $M_2$ as representation vectors, capturing intra-object correlational structure. Concretely, the matrix-objects $X_1$ and $X_2$ are represented as:

$$\begin{aligned} \begin{aligned} V_1&= (-0.16363636, -0.15151515, -0.28484848, -0.04242424, -0.23636364,\\&\quad -0.18787879, -0.18787879, 0.6, 0.49090909, 0.06666667),\\ V_2&= (0.6, 0.2, -0.82857143, 0.37142857, 0.25714286,\\&\quad -0.08571429, -0.08571429, -0.02857143, -0.02857143, -0.54285714). \end{aligned} \end{aligned}$$

Because matrix-objects may contain varying numbers of records, such as $X_1$ and $X_2$, directly defining a distance metric that respects attribute dependencies is nontrivial. Our representation addresses this by mapping every object to a uniform vector form, such as $V_1$ and $V_2$, thereby enabling standard distance-based clustering.

$$\begin{aligned} M_2 = \begin{bmatrix} 1.0 & 0.6 & 0.2 & -0.82857143 & 0.37142857 \\ 0.6 & 1.0 & 0.25714286 & -0.08571429 & -0.08571429 \\ 0.2 & 0.25714286 & 1.0 & -0.02857143 & -0.02857143 \\ -0.82857143 & -0.08571429 & -0.02857143 & 1.0 & -0.54285714 \\ 0.37142857 & -0.08571429 & -0.02857143 & -0.54285714 & 1.0 \end{bmatrix} \end{aligned}$$

### The correlational-structure clustering method

**Fig. 5:**
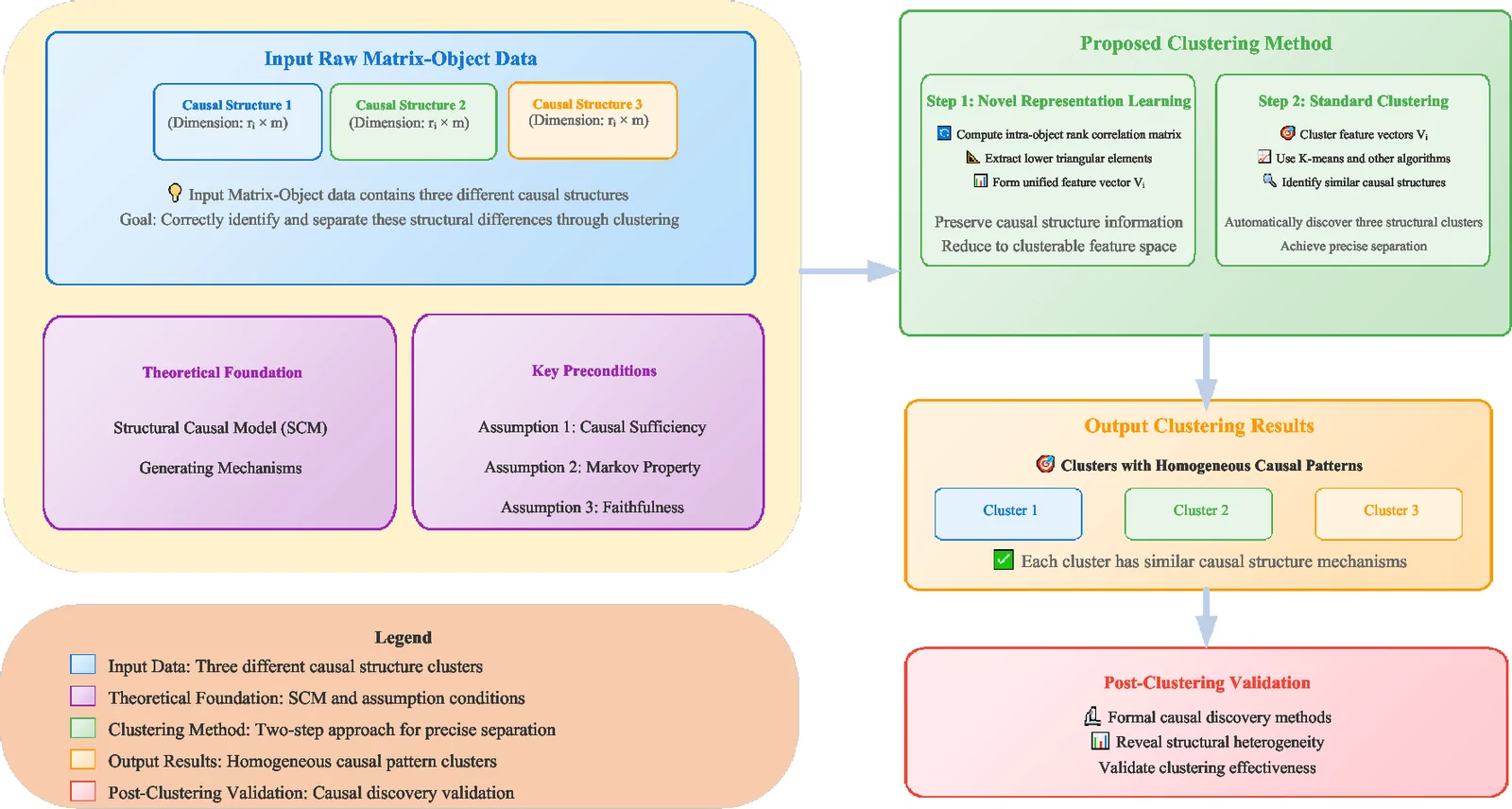
Synthetic matrix-object data is generated based on two SCMs, each of which generates three matrix-objects. The algorithm first maps each matrix-object to an intra-object correlation matrix, then takes the lower triangular part as the new representation, and finally completes clustering by the k-means algorithm.

Building on the representation in Section "A new representation of matrix-object", we propose a correlational-structure clustering method for matrix-object data. First, we compute intra-object rank-based correlations for each matrix-object. Next, Algorithm 1 derives a new representation for each object and forms a dataset, which we cluster with K-means to obtain final labels. Algorithm 2 outlines the full procedure, and Fig. 5 illustrates the complete workflow of our proposed algorithm. Specifically, the left part of the figure shows the input raw matrix-object data generated from distinct SCMs, such as Causal Structure 1, 2 and 3, with each structure producing several matrix-objects. The right-upper part then depicts our two-step clustering method. The first step involves Novel Representation Learning, where we compute the intra-object Spearman rank correlation matrices, extract the lower-triangular elements, and form the unified feature vectors $V_i$. The second step is Standard Clustering, where we apply K-means to these vector representations. Finally, the right-lower part shows the output clustering results, which successfully separates matrix-objects that share similar causal skeletons into homogeneous clusters, thereby avoiding the mixing of heterogeneous regimes prior to any formal causal discovery.

**Algorithm 2:**
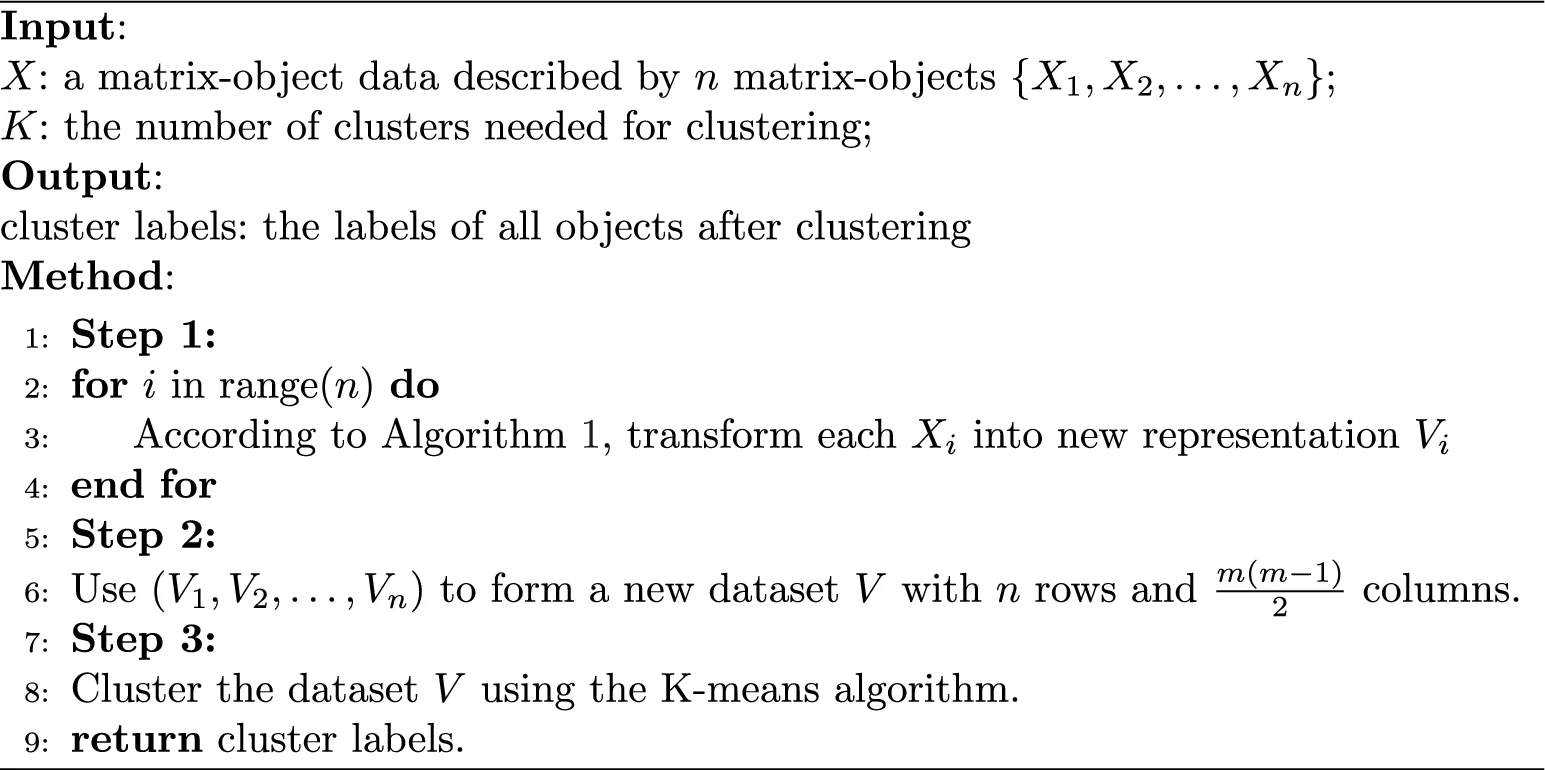
Correlational-structure clustering

**Time Complexity Analysis.** The overall process comprises two main phases: (1) transforming each matrix-object into its new representation, and (2) clustering the resulting representations.

The computational cost of the transformation phase is dominant. Given a dataset of *n* matrix-objects, where each object $X_i$ has $r_i$ records (rows) and *m* features (columns), the algorithm computes an *m × m* rank-based correlation matrix for each object. This requires processing all $\frac{m(m-1)}{2}$ pairs of features. For each feature pair, the $r_i$ observations must be sorted, with a complexity of $O(r_i \log r_i)$. Consequently, the complexity for a single object is $O(m^2 \cdot r_i \log r_i)$, leading to an overall complexity of $O(n \cdot m^2 \cdot r_i \log r_i)$ for the entire dataset.

The subsequent clustering phase operates on the new representation, where each object is represented by a vector of dimension $\frac{m(m-1)}{2}$. Applying the K-means algorithm with *K* clusters for *I* iterations results in a complexity of $O(I \cdot K \cdot n \cdot m^2)$.

Therefore, the total time complexity of the proposed algorithm is the sum of the representation and clustering costs, yielding:

$$\begin{aligned} O\left( n \cdot m^2 \cdot (r_i \log r_i + I \cdot K)\right) . \end{aligned}$$

This analysis highlights that the complexity scales linearly with the number of objects *n*, and quadratically with the number of features *m*, which is typically the dominant factor. The term $r_i \log r_i$, associated with sorting the records per object, becomes significant for objects with a large number of records.

## Experiments

In our experiments, we set the number of clusters *K* to 3 for the synthetic datasets, while for real-world datasets, we determine *K* based on the ground truth (specifically, *K=6* for MOSAD and *K=2* for Mutagenesis1). To generate the synthetic data, we use *n* matrix-objects and *m* features. Here, *n* and *m* are set to 150 and 5 respectively. We also vary the record length *T* across $\{10, 25, 50, 100, 150\}$ to examine its impact. All synthetic experiments are repeated 10 times, and the deep clustering comparisons are repeated 5 times. For the K-means algorithm, we adopt the k-means++ and fix the random seed at 42 to ensure reproducibility.

To validate our method, we design a series of experiments. First, we compare the proposed algorithm against several baselines on both synthetic and real-world datasets. We then analyze how the record count affects the clustering performance. We also examine whether our representation can successfully capture similarities among objects that share the same causal structure. Furthermore, we provide visualizations of the clustering results to illustrate how different regimes are separated. Finally, we offer an empirical justification for using correlation as a proxy for causal skeletons.

### Algorithm comparison

To evaluate the proposed algorithm against established baselines, we perform a comprehensive comparison using two distinct real-world datasets: a multivariate time-series dataset (MOSAD)^31^ and a multi-instance dataset (Mutagenesis1) and synthetic datasets. We benchmark our method against a suite of competitors, including the specialized time-series algorithm TICC (2017)^32^, general-purpose methods like K-means and GMM, and state-of-the-art multi-instance clustering algorithms such as BAMIC, k-Mnv-Rep (2021)^17^, and WMODC (2022)^16^. The subsequent sections will detail these comparisons, demonstrating the robust and superior performance of our approach.

#### Data preprocessing for baseline methods

To ensure reproducible and fair comparisons, we clarify how each baseline processes the input data. Since general-purpose clustering algorithms like K-means and GMM, as well as deep learning approaches such as DEC, IDEC and DCN, cannot handle matrix-objects directly, they require each matrix-object $X_i$ to be flattened into a raw vector of length $r_i \times m$. We apply zero-padding to objects with shorter record lengths so that all vectors share a uniform dimensionality. In contrast, specialized algorithms like BAMIC, k-Mnv-Rep and WMODC take the raw matrix-objects as input directly and rely on their defined dissimilarity measures or representations. Similarly, TICC processes the multivariate time series directly using its Toeplitz inverse covariance formulation without any additional preprocessing steps. For our proposed method, we first compute the Spearman rank correlation matrix for each matrix-object and extract the lower-triangular entries to form a vector of dimension *m(m-1)/2*. We then apply K-means to these correlation vectors.

#### Synthetic dataset setup

Public matrix-object datasets typically do not provide ground-truth causal graphs. To evaluate the effectiveness of the proposed algorithm, we therefore employ synthetic datasets for controlled experiments. Liu et al.^24^ synthesized a time-series dataset to investigate causal discovery in time series. The synthetic dataset was first generated using the Erdős-Rényi model to create random abstract graphs. For each graph, time data were generated using the structural equation:

5 $$\begin{aligned} A_j(t) = \sum _{r \in PA_j} f_{jr}(A_r(t - 1)) + N_j \end{aligned}$$

where the function $f_{jr}$ is randomly chosen from $\{\text {linear}, \text {sin}\}$ and $\{ \text {tanh}, \text {sqrt}\}$, and the exogenous noise $N_j$ is randomly sampled from $\{\text {uniform},\text {Gaussian}\}$ and $\{\text {exponential}, \text {gamma}\}$.

**Fig. 6:**
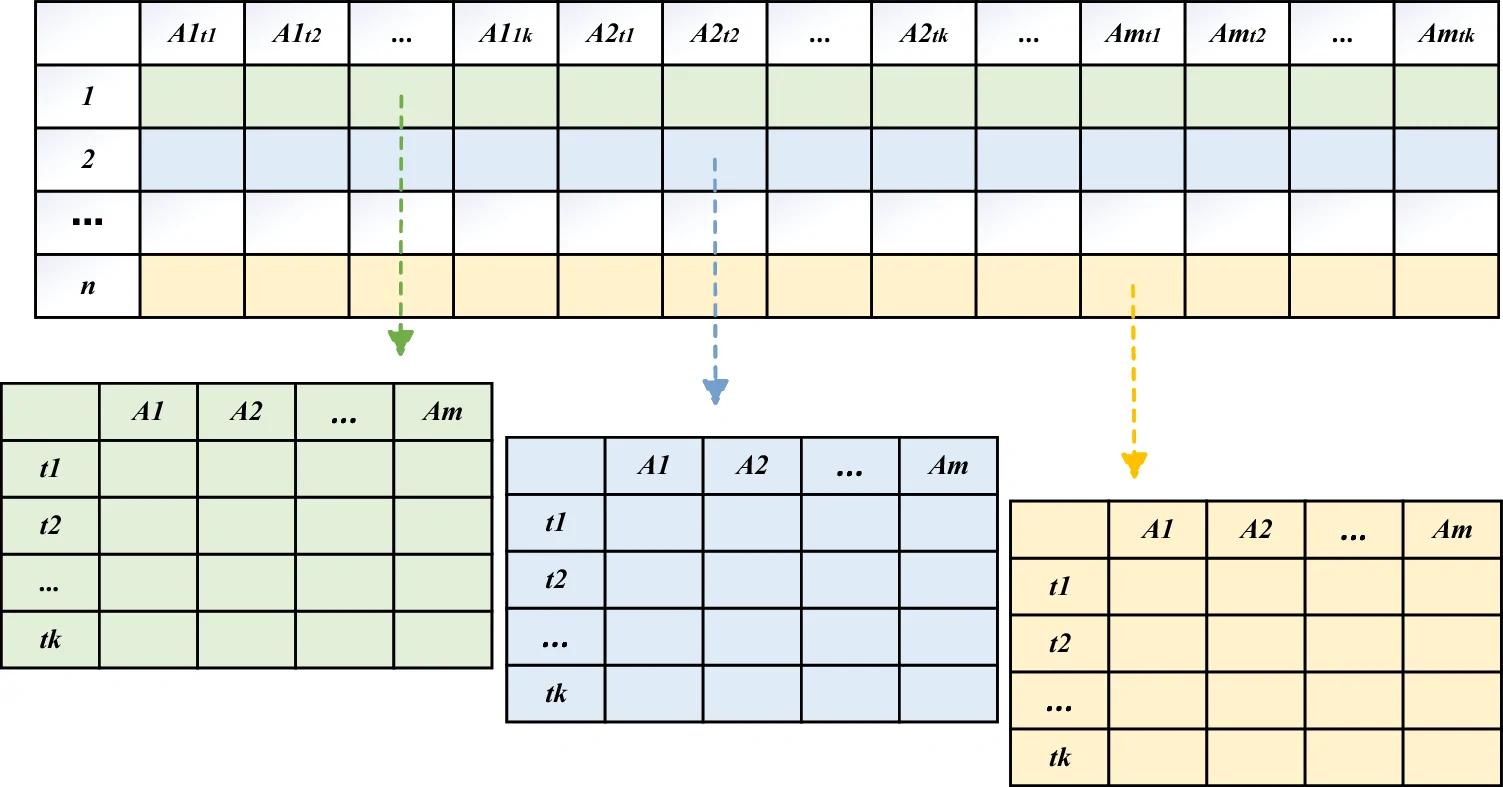
Transform the original raw data, consisting of *n* rows and *m × k* columns, into *n* matrix-objects, where each matrix-object comprises *k* rows and *m* columns.

Inspired by Liu et al.’s method, we generate synthetic matrix-objects as follows (Fig. 6). First, Eq. (5) produces a dataset with *n* objects. Each object contains *k× m* attributes, where $A_{jts}$ denotes the *j*-th attribute at time *s*. We then reshape each object into an *m× k* matrix with rows corresponding to attributes at the same time point, yielding matrix-objects and a matrix-object dataset.

#### Evaluation on synthetic datasets

The data analysis presented in Tables 1 and 5 examined the comparative experimental results when each matrix-object contained 100 records, involving the K-means algorithm, the Bamic algorithm, the WMODC algorithm, and the proposed algorithm. The experimental results indicate that, in each experiment, the Adjusted Rand Index (ARI) and Normalized Mutual Information (NMI) based on the clustering results of the proposed algorithm were significantly superior to those of the compared algorithms.

Table 2 provides the comparative experimental results between the proposed algorithm and the K-means and Bamic algorithms under the condition that each matrix-object contains 50 records. The NMI and ARI values derived from the clustering results of the proposed algorithm were higher than those of the two compared algorithms.

Tables 3 and 4 document the results of 10 comparative experiments conducted when each matrix-object contained 25 and 10 records, respectively. Analysis of these data reveals that the ARI and NMI obtained by the proposed algorithm no longer exhibit a distinct advantage, and in some experiments, the ARI and NMI values of the proposed algorithm were lower than those of the compared algorithms.

**Table 1:** 3 clusters, 150 matrix-objects and 100 records per matrix-object.

	Proposed-ARI	K-Means-ARI	Bamic-ave-ARI	Proposed-NMI	K-means-NMI	Bamic-ave-NMI
1	**0.7195**	0.1465	0.1459	**0.7837**	0.2812	0.2681
2	**0.7853**	0.1647	0.1647	**0.8023**	0.3418	0.3418
3	**0.6722**	0.2236	0.0702	**0.7050**	0.4023	0.1702
4	**0.9030**	0.1650	0.0633	**0.8847**	0.2520	0.1500
5	**0.5307**	0.1353	0.0768	**0.5518**	0.2360	0.2505
6	**0.7988**	0.0636	0.0578	**0.8010**	0.2226	0.1462
7	**0.9799**	0.1628	0.0561	**0.9701**	0.2298	0.2464
8	**0.6788**	0.1354	0.1274	**0.6952**	0.2240	0.2242
9	**0.4670**	0.1881	0.1761	**0.5008**	0.3909	0.3793
10	**0.3985**	0.1111	0.0623	**0.4620**	0.2850	0.2595

**Table 2:** 3 clusters, 150 matrix-objects and 50 records per matrix-object.

	Proposed-ARI	K-Means-ARI	Bamic-ave-ARI	Proposed-NMI	K-Means-NMI	Bamic-ave-NMI
1	**0.6748**	0.1037	0.1133	**0.6918**	0.2750	0.2859
2	**0.7028**	0.1905	0.2039	**0.7277**	0.3057	0.3132
3	**0.3822**	0.1021	0.0864	**0.4102**	0.2743	0.1894
4	**0.3667**	0.1569	0.0541	**0.4381**	0.2229	0.1432
5	**0.9211**	0.0713	0.1335	**0.8895**	0.2332	0.1946
6	**0.4874**	0.1133	0.1574	**0.5583**	0.2859	0.2316
7	**0.6399**	0.1771	0.1771	**0.6821**	0.3540	0.3540
8	**0.9410**	0.1310	0.0898	**0.9192**	0.3071	0.2962
9	**0.7246**	0.1530	0.0657	**0.6930**	0.3301	0.1668
10	**0.7878**	0.3296	0.3296	**0.7483**	0.5064	0.5064

**Table 3:** 3 clusters, 150 matrix-objects and 25 records per matrix-object.

	Proposed-ARI	K-Means-ARI	Bamic-ave-ARI	Proposed-NMI	K-Means-NMI	Bamic-ave-NMI
1	**0.0993**	0.2000	0.0610	0.1056	0.3390	0.1299
2	**0.2823**	0.1231	0.1343	**0.3552**	0.2096	0.2075
3	0.1556	0.1648	0.1530	0.1528	0.3419	0.3301
4	**0.2775**	0.1255	0.0852	**0.2719**	0.2272	0.2051
5	0.2356	0.2417	0.2531	0.2389	0.2992	0.3094
6	**0.4049**	0.0854	0.0542	**0.3985**	0.2533	0.1338
7	**0.2965**	0.0880	0.1633	**0.3143**	0.1991	0.2939
8	**0.2924**	0.0347	0.0188	**0.3777**	0.0858	0.0280
9	**0.5232**	0.1451	0.1985	**0.5542**	0.3198	0.3771
10	**0.2484**	0.1856	0.1850	0.2774	0.3647	0.3644

**Table 4:** 3 clusters, 150 matrix-objects and 10 records per matrix-object.

	Proposed-ARI	K-Means-ARI	Bamic-ave-ARI	Proposed-NMI	K-Means-NMI	Bamic-ave-NMI
1	**0.1689**	0.1332	0.0539	0.1830	0.1897	0.1174
2	**0.1362**	0.1242	0.0306	0.1334	0.2127	0.1100
3	**0.2497**	0.1975	0.0490	0.2877	0.2500	0.0996
4	0.0878	0.1809	0.0573	0.0925	0.2742	0.1893
5	0.0340	0.1275	0.0749	0.0562	0.2988	0.1686
6	**0.2253**	0.1867	0.1663	0.2341	0.3651	0.2588
7	**0.1933**	0.0340	0.0522	0.1810	0.1159	0.0788
8	**0.2506**	0.2102	0.0696	0.3229	0.3893	0.1904
9	0.1112	0.2003	0.0905	0.1539	0.3144	0.1716
10	**0.1851**	0.0561	0.0462	**0.1753**	0.1396	0.1405

**Table 5:** 3 clusters, 150 matrix-objects and 100 records per matrix-object.

	Proposed-ARI	WMODC-ARI	Proposed-NMI	WMODC-NMI
1	**0.4094**	0.0296	**0.5660**	0.0366
2	**0.4893**	−0.0003	**0.5582**	0.0131
3	**0.8263**	−0.0031	**0.8215**	0.0096
4	**0.6618**	0.0049	**0.7092**	0.0156
5	**0.5531**	0.0061	**0.5496**	0.0231
6	**0.7853**	−0.0043	**0.8023**	0.0079
7	**0.8849**	−0.0086	**0.8701**	0.0034
8	**0.5789**	−0.0066	**0.7106**	0.0082
9	**0.6026**	−0.0048	**0.6437**	0.0069
10	**0.9410**	−0.0038	**0.9306**	0.0091

#### Evaluation on real-world datasets

**Fig. 7:**
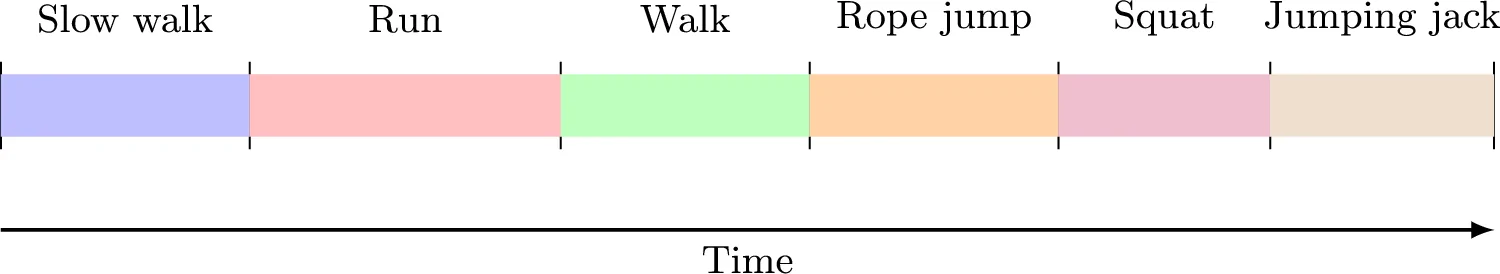
Schematic timeline of six activity states in the real multivariate time series; colored segments indicate contiguous regimes. Segmentation yields matrix-objects used for clustering.

We convert multivariate time series into matrix-objects via segmentation. MOSAD (Mobile Sensing Human Activity Data Set)^31^ is a multi-modal, annotated dataset collected from 6 participants across 3 motion sequences. We select records from one participant in the third sequence, which includes six activity states: slow walk, run, walk, rope jump, squat, and jumping jack—thus the time series comprises six distinct activity regimes. Time-series segmentation produces matrix-objects used to assess our clustering method. If matrix-objects belong to the same activity state, their intra-object correlational structures should be similar. Fig. 7 provides an illustrative timeline of the six activity states (slow walk, run, walk, rope jump, squat, jumping jack) observed in the real multivariate time series. Each colored segment denotes a contiguous regime in time; segmentation produces matrix-objects accordingly for clustering and subsequent analysis.

**Fig. 8:**
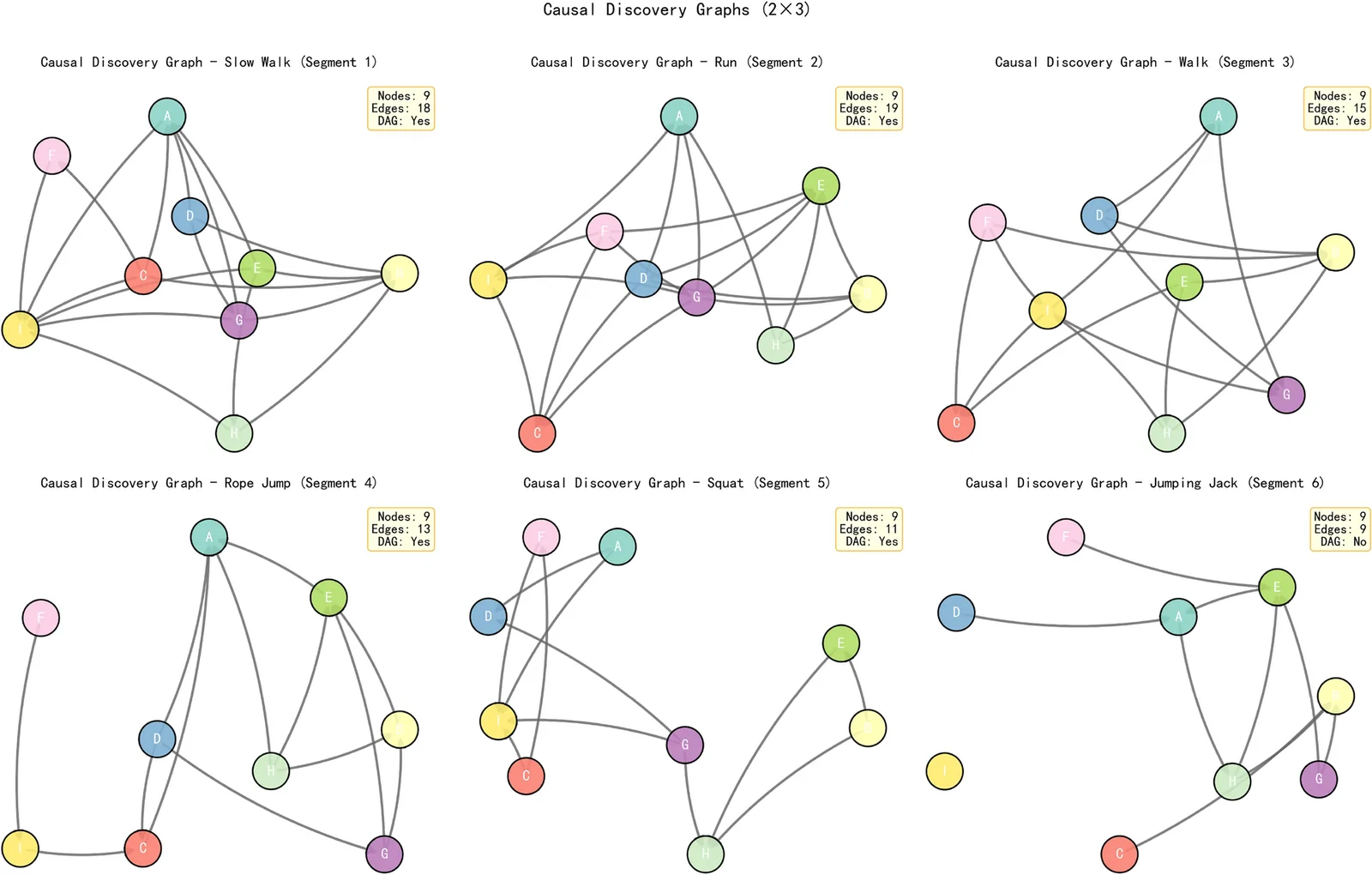
Post-clustering causal discovery graphs for six activity states in the real multivariate time-series demonstration; regime-specific differences substantiate that each activity state corresponds to a distinct causal skeleton.

**Fig. 9:**
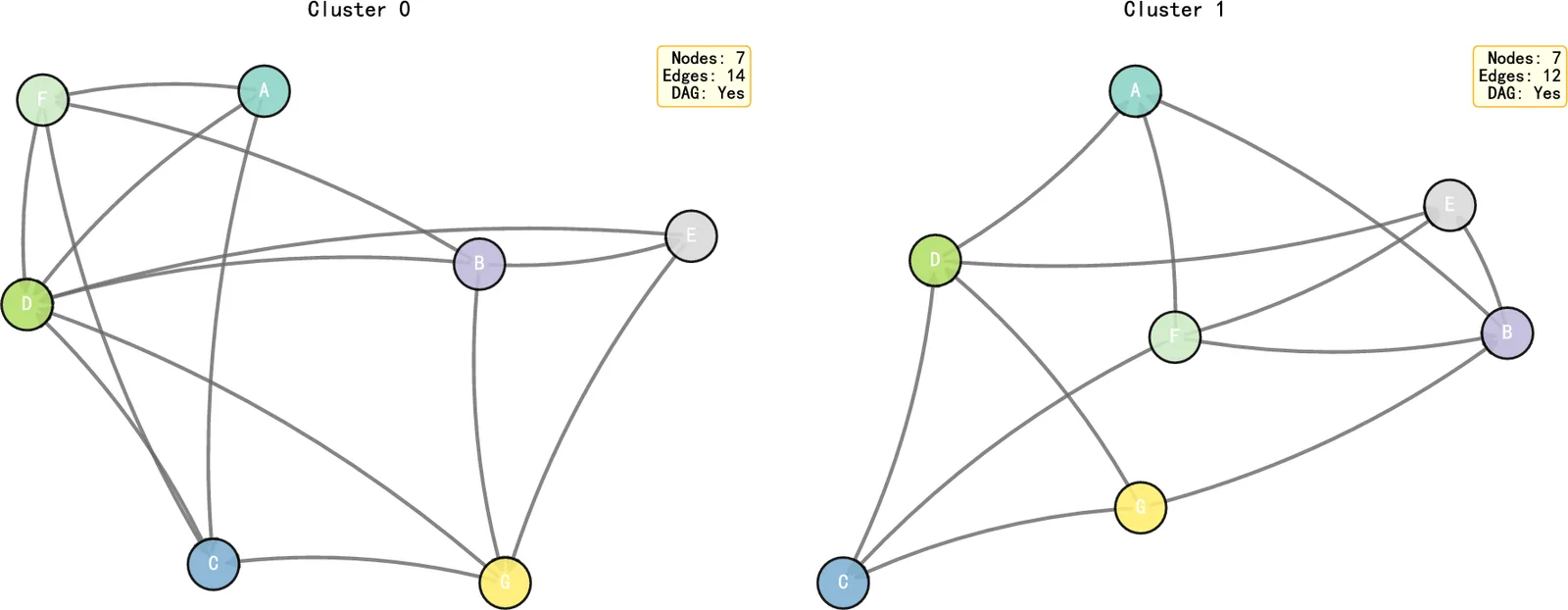
Causal discovery graphs for Mutagenesis1 (K=2) clustering results.

As a post-clustering verification step, we conduct formal causal discovery on the clusters identified in our real-data experiments. For the multivariate time-series data, the resulting causal graphs for the six activity regimes are presented in Fig. 8. For the Mutagenesis1 dataset, the graphs for the two identified clusters are shown in Fig. 9. In both cases, the graphs exhibit clear regime-specific structural heterogeneity, indicating that the clusters correspond to distinct causal skeletons. This corroborates that our clustering approach successfully separates structurally different groups, while adhering to our stated boundary that causal claims require explicit discovery and expert validation.

In addition to visualization, we compare our method against established baselines on the real dataset and the Mutagenesis1 multi-instance dataset.

We evaluate our method on the real-data comparison. The clustering performance against specialized and general baselines is summarized alongside results in Table 6.

**Table 6:** Comparison on real-world datasets and Mutagenesis1 datasets (ARI/NMI). Missing evaluations are denoted by “-”.

Method	MOSAD-ARI	MOSAD-NMI	Muta-ARI	Muta-NMI
Proposed	**0.9244**	**0.9423**	**0.1682**	**0.1533**
TICC	0.5393	0.7219	0.0970	0.0748
GMM	0.3924	0.5708	0.0812	0.1232
K-Means	0.3473	0.5094	0.1258	0.0945
Bamic-ave	0.3934	0.6046	−0.0023	0.0082
k-Mnv-Rep	-	-	0.1340	0.1145
WMODC	0.3015	0.5357	0.1381	0.1194

### Effect of record count per matrix-object on algorithm performance

In this section, we examine how record counts per matrix-object affect the proposed algorithm. Table 7 reports average ARI and NMI over 10 runs with 10, 25, 50, 100, and 150 records.

**Fig. 10:**
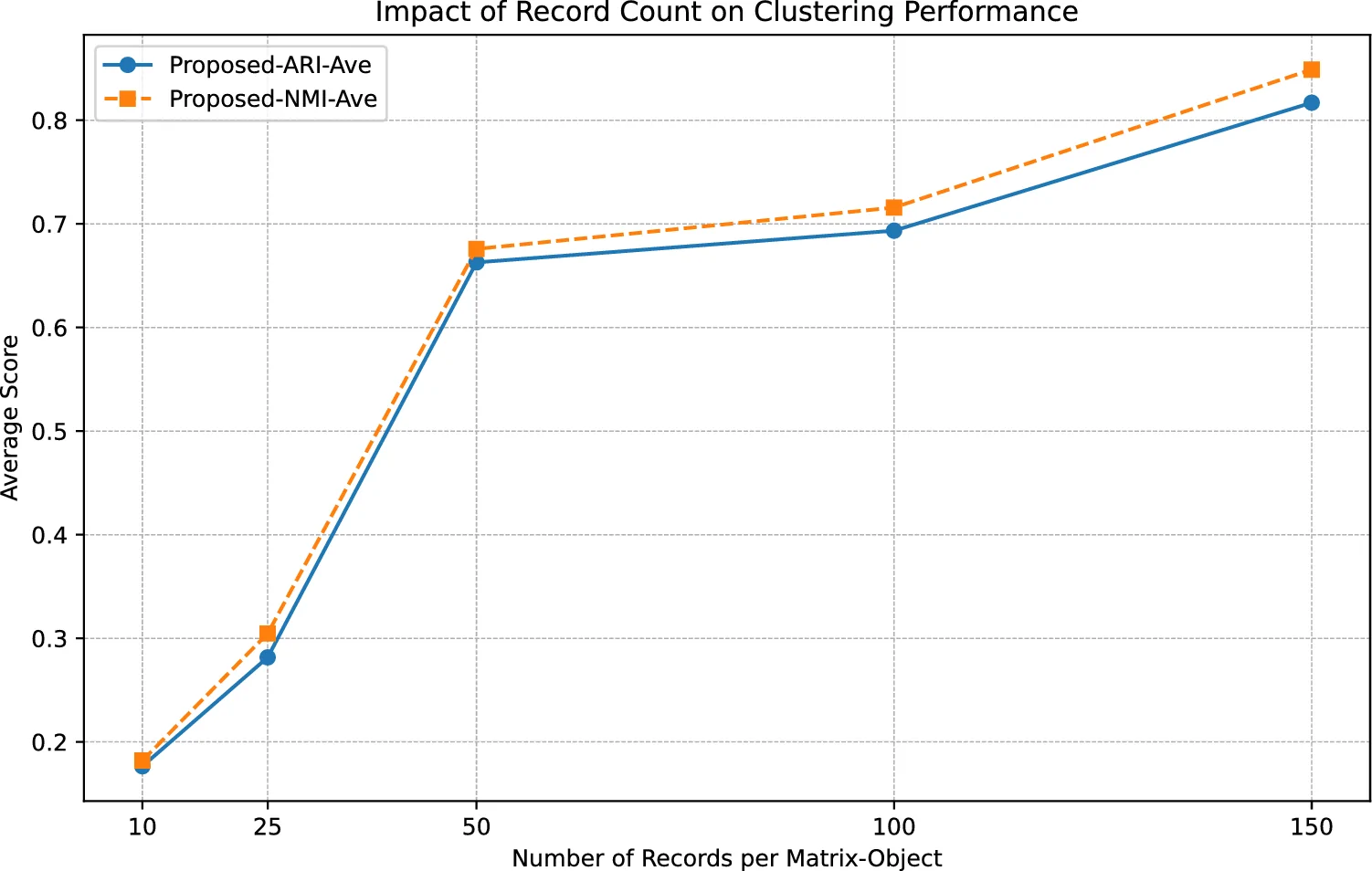
Impact of record count on the proposed algorithm.

Fig. 10 reveals a relationship between record count and clustering performance: as the number of records decreases, clustering accuracy diminishes. With more records per matrix-object, the representation is estimated more precisely and better reflects intra-object correlational structure.

**Table 7:** Average NMI and ARI of the proposed algorithm under varying record counts.

	Proposed-ARI-Ave	Proposed-NMI-Ave
150	0.8169	0.8489
100	0.6934	0.7157
50	0.6628	0.6758
25	0.2816	0.3046
10	0.1765	0.1820

### Representation similarity of matrix-objects under shared and distinct causal structures

Euclidean distance is a standard metric used in clustering. For two points $\textbf{p} = (p_1, p_2, \ldots , p_n)$ and $\textbf{q} = (q_1, q_2, \ldots , q_n)$ in *n*-dimensional space, the distance is defined as:

6 $$\begin{aligned} d(\textbf{p}, \textbf{q}) = \sqrt{\sum _{i=1}^{n} (p_i - q_i)^2} \end{aligned}$$

**Fig. 11:**
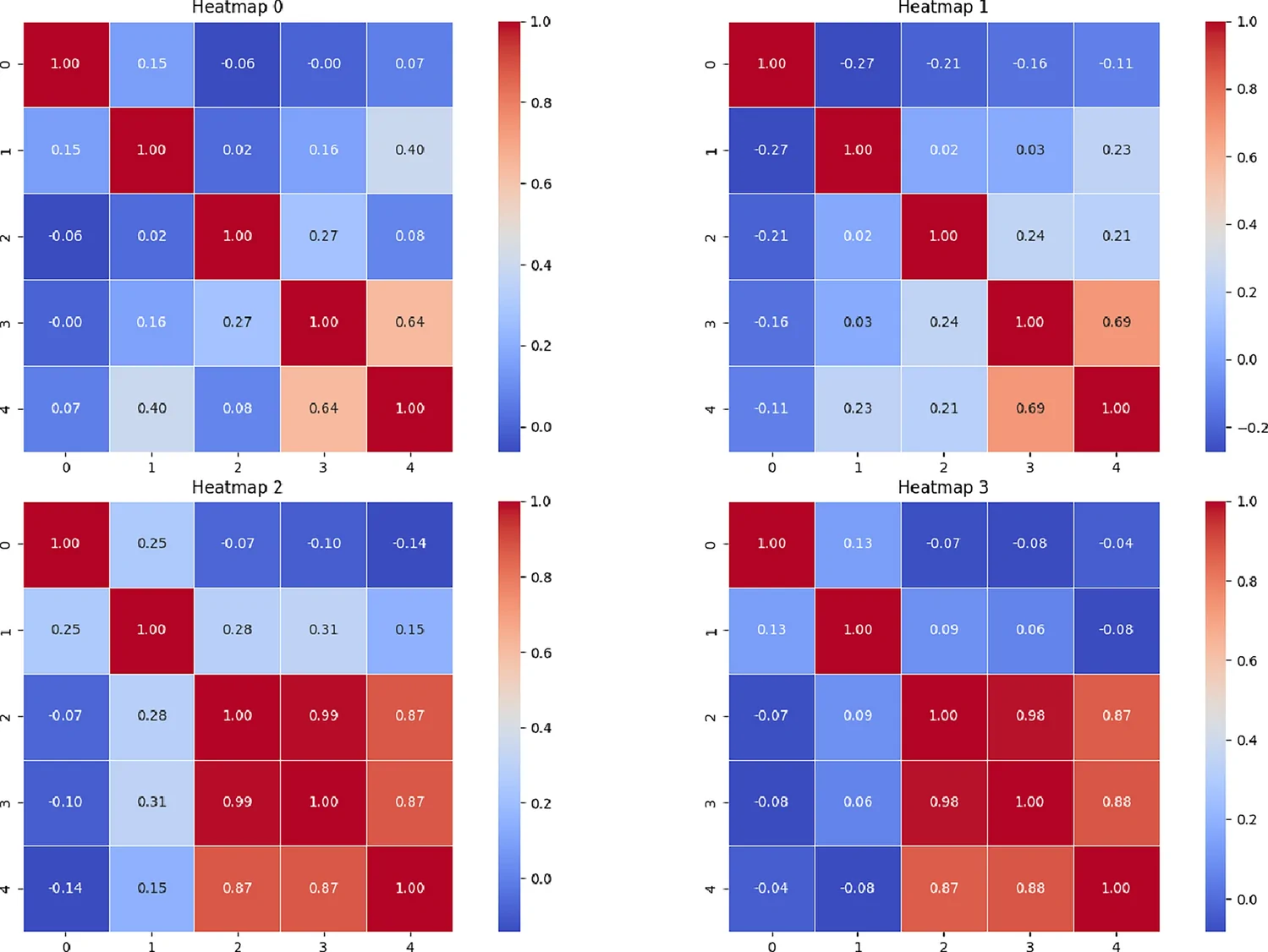
Representation of matrix-object heatmap.

We synthesize datasets based on the Temporal Structural Model (TSM), comprising four matrix-objects, each containing five features and 100 records. To investigate the similarities in the representation of matrix-objects sharing the same causal structure—and, in synthetic data where generating SCM regimes are known, alignment with those regimes—we perform an exploratory analysis shown in Fig. 11.

As depicted in Fig. 11, Heatmap 0 and Heatmap 1 originate from the same TSM, while Heatmap 2 and Heatmap 3 stem from another identical TSM. For the four matrices in Fig. 11, the Euclidean distance between Heatmap 0 and Heatmap 1 (using the lower-triangular entries) is 0.56, and between Heatmap 2 and Heatmap 3 is 0.42. The distances from Heatmap 0 to Heatmaps 2 and 3 are 1.18 and 1.21, respectively; from Heatmap 1 to Heatmaps 2 and 3 they are 1.22 and 1.14. These distances indicate that representations derived from the same TSM are closer.

### Visualization: original distributions versus clustering outcomes

We compare empirical distributions with clustering outcomes across three synthesized scenarios with $K\!\in \!\{3,4,5\}$ and 150 points per cluster. The top panels depict unlabeled original distributions, while the bottom panels show cluster assignments. Points are drawn around distinct centers with small random jitter to emulate well-separated regimes. The results recover the underlying group structure and yield clear regime separation.

**Fig. 12:**
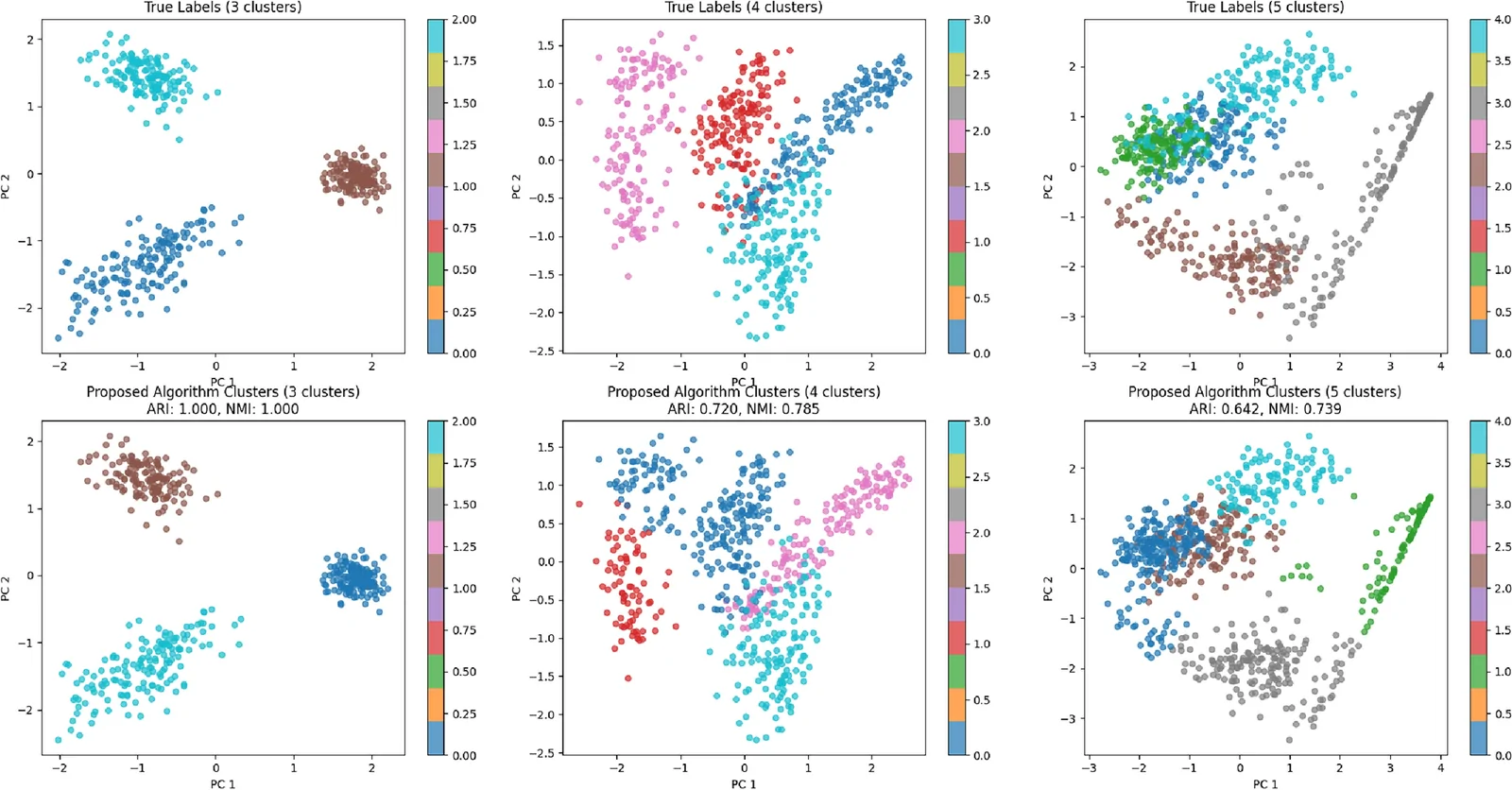
Visualization of Large-Scale Clustering Results (150 Records per Matrix-Object).

**Fig. 13:**
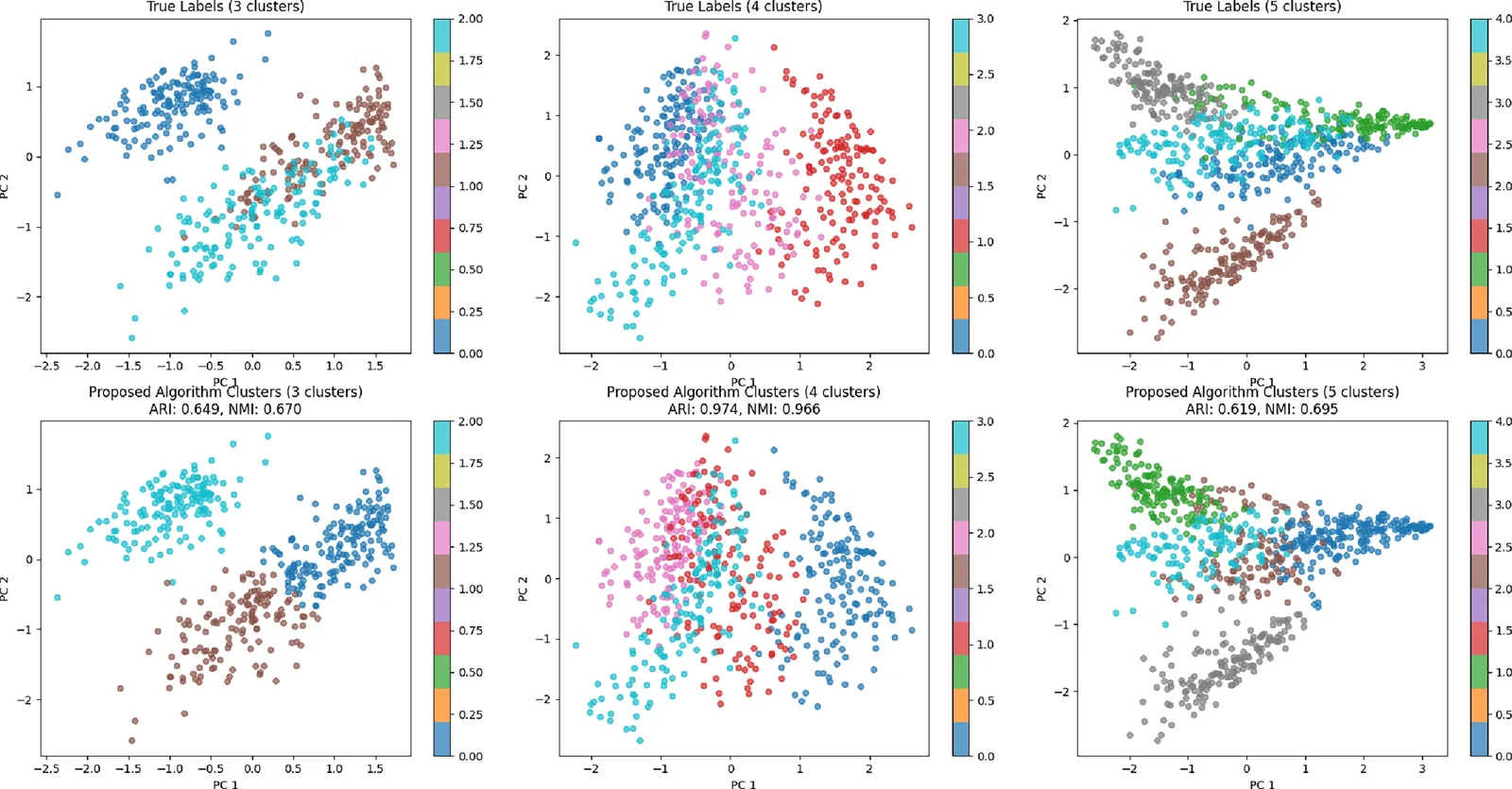
Visualization of Large-Scale Clustering Results (50 Records per Matrix-Object).

We provide a large-scale visualization with 150 records per matrix-object, demonstrating stable separation of correlational structures when record counts are high (Fig. 12). Additionally, we consider a setting with 50 records per matrix-object to illustrate stability when object counts per cluster are high and records are moderate (Fig. 13).

### Justification of correlation as a proxy for causal skeletons

To clarify why correlation does not imply causation—and why we treat correlational patterns as proxy signals only—we simulate four regimes: confounding (Z*→*X, Z*→*Y), collider with selection (X*→*Z*←*Y, with selection on high Z), direct causal (X*→*Y), and independence. With 200,000 samples per regime, the observed correlations are: confounding $\textrm{corr}(X,Y)=0.799$; collider overall $\textrm{corr}(X,Y)=0.004$; collider under selection $\textrm{corr}(X,Y)=-0.527$; direct causal $\textrm{corr}(X,Y)=0.894$; independence $\textrm{corr}(X,Y)=-0.001$. These results show that high correlation can arise without causation (confounding), that selection on a collider can induce strong spurious correlation (negative in this case), that genuine causal influence yields high correlation, and that independence yields near-zero correlation. Accordingly, our clustering uses correlational structures only as proxy signals—under stated assumptions—to avoid mixing heterogeneous regimes; causal claims require post-clustering causal discovery and expert validation.

**Table 8:** Empirical stress test: correlations across four regimes (N=200,000).

Regime	corr(X,Y)
Confounding (Z*→*X, Z*→*Y)	0.7990
Collider overall (X*→*Z*←*Y)	0.0040
Collider selected (Z high)	−0.5270
Direct causal (X*→*Y)	0.8940
Independent baseline	−0.0010

### Ablation studies

To further validate the design choices of our method and address potential concerns, we conduct a comprehensive set of ablation studies covering correlation type selection, clustering algorithm choice, feature dimension scalability, assumption robustness, small-sample estimation, and comparison with deep clustering methods.

#### Correlation type ablation

A natural question is why Spearman’s rank correlation is chosen over other correlation measures. We compare Spearman with Pearson correlation under varying record lengths. Table 9 shows that when *T ≥ 50*, Spearman consistently outperforms Pearson, while for very short records (*T < 50*), the two are comparable. This confirms that Spearman’s ability to capture monotone (not just linear) dependencies provides a meaningful advantage for our proxy-signal framework.

**Table 9:** Ablation study on correlation type: Spearman vs. Pearson under varying record lengths (*m=5*, 3 clusters). Best results are in bold.

*T*	Spearman		Pearson	
	ARI	NMI	ARI	NMI
10	*0.195 ± 0.092*	*0.219 ± 0.098*	$\mathbf {0.223 \pm 0.122}$	$\mathbf {0.248 \pm 0.126}$
25	*0.357 ± 0.131*	*0.390 ± 0.119*	$\mathbf {0.377 \pm 0.132}$	$\mathbf {0.403 \pm 0.121}$
50	$\mathbf {0.652 \pm 0.181}$	$\mathbf {0.662 \pm 0.159}$	*0.641 ± 0.191*	*0.659 ± 0.167*
100	$\mathbf {0.756 \pm 0.184}$	$\mathbf {0.775 \pm 0.174}$	*0.746 ± 0.189*	*0.767 ± 0.180*
150	$\mathbf {0.850 \pm 0.189}$	$\mathbf {0.862 \pm 0.166}$	*0.835 ± 0.193*	*0.851 ± 0.173*

#### Alternative clustering algorithms

Since K-means assumes Euclidean geometry and convex clusters, we compare it with six alternative clustering algorithms on the Spearman correlation vectors. Table 10 reports results at *T=100*, and Table 11 shows the comparison across varying record lengths. Spectral-NN achieves the best performance, followed by GMM and K-means. At *T ≥ 100*, Spectral-NN is the strongest; at moderate *T*, GMM performs best. These results suggest that while alternative algorithms can provide marginal improvements, the quality of the Spearman representation itself is the dominant factor.

**Table 10:** Comparison of clustering algorithms on Spearman correlation vectors (*m=5*, *T=100*, 3 clusters). Best results are in bold.

Method	ARI	NMI
K-Means	0.709±0.200	0.722±0.157
GMM	0.865±0.141	0.873±0.122
Spectral-RBF	0.641±0.269	0.691±0.220
Spectral-NN	**0.874±0.184**	**0.899±0.120**
Agg-Ward	0.765±0.221	0.809±0.168
Agg-Average	0.443±0.152	0.593±0.148
DBSCAN	0.680±0.145	0.731±0.100

**Table 11:** Clustering algorithm comparison under varying record lengths (*m=5*, 3 clusters). Best ARI per row is in bold.

*T*	K-Means		GMM		Spectral-NN		Agg-Ward	
	ARI	NMI	ARI	NMI	ARI	NMI	ARI	NMI
10	0.157	0.168	0.157	0.168	0.133	0.163	0.132	0.151
25	0.393	0.402	0.426	0.439	0.339	0.386	0.367	0.392
50	0.660	0.694	**0.801**	**0.804**	0.747	0.769	0.635	0.682
100	0.687	0.733	0.734	0.757	**0.761**	**0.804**	0.669	0.735
150	0.743	0.783	0.829	0.858	**0.880**	**0.888**	0.726	0.777

#### Feature dimension scalability

To assess the effect of increasing feature dimension *m*, we vary $m \in \{3, 5, 10\}$ and report both clustering quality and runtime in Table 12. As *m* increases, the representation dimension grows as *m(m-1)/2*, yet clustering quality improves substantially.For example, the value of ARI is from 0.458 to 0.877, when m is from 3 to 10. That confirms that richer correlational structure provides stronger discriminative signals. Runtime scales modestly from 0.09s to 1.18*s*, demonstrating practical efficiency. Additionally, Table 13 shows that PCA dimensionality reduction to 15 dimensions slightly improves performance for *m=10*, suggesting that denoising via PCA can be beneficial in high-dimensional settings.

**Table 12:** Clustering performance under varying feature dimension *m* (*T=100*, 3 clusters). The vector dimension grows as *m(m-1)/2*.

*m*	Vec. Dim.	ARI	NMI	Runtime (s)
3	3	0.458±0.121	0.513±0.098	0.09±0.01
5	10	0.769±0.191	0.806±0.160	0.27±0.01
10	45	0.877±0.110	0.891±0.083	1.18±0.02

**Table 13:** Effect of PCA dimensionality reduction on clustering performance (*T=100*, 3 clusters).

*m*	NoPCA ARI	NoPCA NMI	PCA*→*15 ARI	PCA*→*15 NMI	PCA*→*20 ARI	PCA*→*20 NMI
10	0.863±0.213	0.882±0.136	0.889±0.169	0.915±0.113	0.890±0.172	0.918±0.115

#### Assumption violation robustness

As outlined in Section "A new representation of matrix-object", our method relies on three key assumptions. To evaluate their impact, we systematically violate each assumption and measure the resulting changes in clustering quality. Table 14 reports the ARI and NMI under three distinct violation scenarios. First, we test the non-monotone mechanism ratio by replacing a fraction of the structural functions with non-monotone alternatives. Second, we assess hidden confounding strength by introducing unobserved common causes. Third, we examine noise correlation by making the exogenous noise terms correlated. The results demonstrate that our method is robust to hidden confounding and noise correlation, with performance remaining stable or even slightly improving under moderate violations. In contrast, non-monotone mechanisms cause the most significant degradation. Specifically, as the non-monotone ratio increases from 0 to 1.0, the ARI drops from 0.674 to 0.548. This is expected since Spearman correlation specifically captures monotone dependencies. This finding highlights the primary limitation of our approach and suggests that exploring non-monotone dependency measures could be a valuable extension.

**Table 14:** Robustness of clustering performance under assumption violations (*m=5*, *T=100*, 3 clusters).

Level	Non-monotone Ratio		Confounder Strength		Noise Correlation	
	ARI	NMI	ARI	NMI	ARI	NMI
0.0	0.674±0.254	0.738±0.192	0.768±0.231	0.806±0.177	0.724±0.259	0.781±0.199
0.25	0.757±0.221	0.808±0.152	0.618±0.137	0.687±0.100	0.738±0.133	0.792±0.102
0.5	0.601±0.307	0.676±0.248	0.731±0.197	0.782±0.135	0.798±0.241	0.840±0.179
0.75	0.576±0.212	0.644±0.164	0.740±0.208	0.770±0.171	0.825±0.216	0.858±0.155
1.0	0.548±0.214	0.613±0.180	0.705±0.279	0.739±0.241	-	-

#### Small-sample shrinkage estimators

When the number of records per matrix-object is small, correlation estimates can be unreliable. We therefore evaluate whether shrinkage estimators can stabilize the correlation matrix. Table 15 compares the raw Spearman correlation with Ledoit-Wolf, Oracle Approximating Shrinkage, and Graphical LASSO under varying record lengths. The Ledoit-Wolf estimator provides marginal improvements at *T=25* and *T=50*. For example, the ARI increases from 0.379 to 0.390 at *T=25*. However, these gains diminish when *T* is 150. Additionally, Graphical LASSO performs poorly in our low-dimensional setting. These results suggest that while shrinkage estimators offer modest benefits for very small samples, the raw Spearman correlation itself is already reasonably robust.

**Table 15:** Effect of shrinkage estimators on clustering performance under varying record lengths (*m=5*, 3 clusters). Best results per row are in bold.

*T*	Raw Spearman		Ledoit-Wolf		OAS	
	ARI	NMI	ARI	NMI	ARI	NMI
10	**0.138**±0.067	**0.142**±0.063	0.127±0.058	0.138±0.059	0.100±0.051	0.115±0.056
25	0.379±0.150	**0.408**±0.126	**0.390**±0.154	0.408±0.131	0.307±0.113	0.340±0.104
50	0.640±0.184	0.661±0.151	**0.644**±0.184	**0.664**±0.152	0.631±0.176	0.651±0.146
150	**0.865**±0.167	**0.897**±0.127	0.865±0.167	0.897±0.127	0.862±0.170	0.895±0.130

#### Comparison with deep clustering methods

Deep clustering methods learn representations end-to-end from raw data. We compare our method with three representative deep clustering algorithms: DEC^33^, IDEC^34^ and DCN^35^. These approaches take flattened raw matrices as input and jointly optimize embedding learning and clustering. Table 16 shows that our method significantly outperforms all deep clustering approaches. Specifically, our method achieves an ARI of 0.692, whereas the deep methods range from only 0.007 to 0.116. Even K-means applied to raw flattened data yields an ARI of 0.143, outperforming the deep methods. This demonstrates that domain-knowledge-driven feature engineering via Spearman correlation is far more effective than end-to-end deep learning for this task. The primary reason is that flattening the matrix discards the critical correlational structure between features, which our method explicitly captures.

**Table 16:** Comparison with deep clustering methods (*m=5*, *T=100*, 3 clusters). Our method uses Spearman correlation as structural representation, while deep methods learn embeddings end-to-end from raw flattened matrices.

Method	ARI	NMI	Time (s)
Proposed	**0.692**±**0.212**	**0.736**±**0.184**	0.34
DEC	0.110±0.049	0.173±0.041	1.17
IDEC	0.116±0.092	0.204±0.136	0.82
DCN	0.007±0.011	0.023±0.012	1.46
KMeans	0.143±0.066	0.247±0.095	0.05

## Conclusions, discussions, and future work

### Conclusions

We introduced a simple, interpretable clustering approach for matrix-object data that groups objects according to their intra-object correlational structure. This structure serves as a proxy for underlying causal signals, enabling regime separation before any formal causal discovery is performed. By converting each matrix-object into a unified representation that captures feature-level dependency patterns, our method supports clustering with standard distance measures while maintaining interpretability.

The performance of the method was rigorously evaluated through extensive synthetic and real-world experiments. The results demonstrate that our method consistently achieves competitive or superior ARI and NMI scores under varied conditions. On the MOSAD multivariate time-series dataset, our approach delivered excellent results (ARI=0.9244, NMI=0.9423), substantially outperforming both specialized and general clustering algorithms. On the more challenging Mutagenesis 1 multi-instance dataset, it also attained the highest performance among state-of-the-art competitors (NMI=0.1533, ARI=0.1682). Furthermore, we conducted post-clustering verification via formal causal discovery on real data. The resulting regime-specific causal graphs exhibit clearly distinct skeletons, corroborating that the identified clusters reflect genuine structural heterogeneity.

### Practical guidance and applicability

To assist researchers in assessing whether our method is appropriate for their data, we provide practical guidance on when the approach is likely to benefit, or potentially mislead—causal analysis.

**When a researcher is likely to benefit:** Our method performs well when matrix-object data has an adequate record length of *T ≥ 50* for stable correlation estimation. As shown in Table 9, the ARI reaches at least 0.652 at *T=50*. The approach is also suitable for domains where monotone mechanisms are plausible, such as economic indicators, biological dose-response relationships, or physical systems with monotone input-output dynamics. Additionally, it is valuable for exploratory causal analysis, helping to identify subgroups that likely share causal mechanisms before conducting expensive or time-consuming causal discovery. Another strength is regime detection, such as in finance, climate science, or industrial monitoring, where different operational regimes correspond to distinct causal structures.

**When a researcher might be led astray:** Our approach can be misleading if the relationships in the data are predominantly non-monotone patterns, such as periodic or threshold functions. Spearman correlation often fails to capture these types of dependencies, which Table 14 illustrates as the ARI drops from 0.674 to 0.548 under non-monotone violations. Very short record lengths, specifically *T < 25*, can also lead to misleading results due to unstable correlation estimates regardless of the true causal structure. Finally, it is crucial not to interpret the resulting clusters as definitive causal groups, but rather as hypotheses for subsequent causal analysis. Two objects might exhibit similar correlation patterns for non-causal reasons like measurement artifacts, meaning these clusters should serve as input for formal causal discovery rather than substitutes.

### Epistemological reflections

Our method implies a layered epistemology of causation that merits explicit discussion:

- **Layer 1—Correlational patterns as observable manifestations.** Correlations are the empirical layer—what we can observe and measure. They are the shadows cast by causal structures on the data.
- **Layer 2—Causal skeletons as latent structures.** The causal skeleton (the set of dependency relationships) is the latent layer—it exists but is not directly observable. Under our assumptions, the correlational pattern is a faithful projection of the causal skeleton onto the observable space.
- **Layer 3—Causal directionality as deeper structure.** The direction of causal effects is an even deeper layer that cannot be recovered from correlations alone. Our method operates at Layers 1–2 and deliberately stops short of Layer 3.

### Limitations and future work

Several limitations of the current approach should be noted. First, performance is sensitive to the number of records per object, as scarce data can lead to unreliable dependency estimates. As demonstrated in Table 15, while we recommend Ledoit-Wolf shrinkage as a practical enhancement for small-sample scenarios, alternative strategies like Bayesian correlation estimation or domain-knowledge-informed priors may be necessary for extremely small samples where *T ≤ 10*. Second, the primary limitation lies in the reliance on monotone mechanisms; when structural functions are predominantly non-monotone, the discriminative power of Spearman correlation degrades. Third, the computational complexity scales quadratically with the number of features to an order of $O(n \cdot m^2 \cdot (r_i \log r_i + I \cdot K))$, rendering the method most suitable for moderate-dimensional settings.

Future work will focus on mitigating these limitations. A key direction is to extend beyond rank correlations to incorporate more powerful similarity measures, such as distance correlation or mutual information, capable of capturing complex and non-linear dependencies without sacrificing interpretability. We also plan to develop a principled strategy for automatically selecting the number of clusters *K*, possibly using stability-based criteria or Bayesian nonparametric models. Finally, extending the framework to nonstationary and online settings will be essential for real-world applications involving dynamically evolving regimes.

**Boundary statement.** Correlation reveals the footprint of causation, not its direction. In our workflow, correlational structures are used only as proxy signals under stated assumptions to separate regimes prior to any formal causal discovery. Any causal claims should be made only after post-clustering causal discovery, domain knowledge integration, and sensitivity analyses.

## Data Availability

All datasets analyzed in this article have been published and are available on public websites. All data used in this article can be downloaded. The download address for the MOSAD dataset is The download address for the MOSAD dataset is https://github.com/ermshaua/mobile-sensing-human-activity-data-set. The download address for the Mutageniss1 dataset is https://www.kaggle.com/datasets/keilacamarillo/mutagenesis-dataset.
